# Piloting HealthScore: Feasibility and acceptability of a clinically integrated health coaching program for people living with cancer

**DOI:** 10.1002/cam4.5625

**Published:** 2023-01-16

**Authors:** William A. Wood, Carly Bailey, Brianna Castrogivanni, Diana Mehedint, Ashley Leak Bryant, Kyle Lavin, Xianming Tan, Jaime Richardson, Yiqing Qian, Kelly R. Tan, Erin E. Kent

**Affiliations:** ^1^ Lineberger Comprehensive Cancer Center University of North Carolina at Chapel Hill Chapel Hill North Carolina USA; ^2^ Department of Medicine, School of Medicine University of North Carolina at Chapel Hill Chapel Hill North Carolina USA; ^3^ Department of Medicine Duke University School of Medicine Durham North Carolina USA; ^4^ School of Nursing University of North Carolina at Chapel Hill Chapel Hill North Carolina USA; ^5^ Department of Psychiatry, School of Medicine University of North Carolina at Chapel Hill Chapel Hill North Carolina USA; ^6^ Department of Biostatistics, Gillings School of Global Public Health University of North Carolina at Chapel Hill Chapel Hill North Carolina USA; ^7^ Department of Health Behavior, Gillings School of Global Public Health University of North Carolina at Chapel Hill Chapel Hill North Carolina USA; ^8^ Department of Health Policy and Management, Gillings School of Global Public Health University of North Carolina at Chapel Hill Chapel Hill North Carolina USA

**Keywords:** cancer, health coaching, physical function, pilot, self‐efficacy, symptom management, telehealth

## Abstract

**Background:**

Cancer supportive care interventions often have limited generalizability, goal misalignment, and high costs. We developed and piloted a health coaching intervention, UNC HealthScore, in patients undergoing cancer treatment (ClinicalTrials.gov identifier NCT04923997). We present feasibility, acceptability, and preliminary outcome data.

**Methods:**

HealthScore is a six‐month, theory‐based, multicomponent intervention delivered through participant‐driven coaching sessions. For the pilot study, participants were provided a Fitbit, responded to weekly symptom and physical function digital surveys, and met with a health coach weekly to develop and monitor goals. Coaching notes were discussed in weekly interdisciplinary team meetings and provided back to the treating oncology team. Symptom alerts were monitored and triaged through a study resource nurse to relevant supportive care services. Feasibility was determined based on intervention enrollment and completion. Acceptability was based on satisfaction with coaching and Fitbit‐wearing and was informed by semistructured exit interviews. Outcomes evaluated for signs of improvement included several PROMIS (Patient‐Reported Outcomes Measurement Information System) measures, including the primary intervention target, physical function.

**Results:**

From May 2020 to March 2022, 50 participants completed the single‐arm pilot. Feasibility was high: 66% enrolled and 71% completed the full intervention. Participants reported an average of 4.8 and 4.7 (out of 5) on the acceptability of coaching calls and using the Fitbit, respectively. Physical function scores rose 3.1 points (SE = 1.1) from baseline to 3 months, and 4.3 (SE = 1.0) from baseline to 6 months, above established minimal clinically important difference (MCID). Improvements above MCID were also evident in anxiety and depression, and smaller improvements were demonstrated for emotional support, social isolation, cognitive function, symptom burden, and self‐efficacy.

**Discussion:**

HealthScore shows feasibility, acceptability, and promising preliminary outcomes. Randomized studies are underway to determine the efficacy of preserving physical function in patients with advanced cancer.

## Graphical abstract

Figure 1 depicts the HealthScore clinically integrated coaching program for patients with cancer. Each component of health coaching program, including monitoring (weekly patient‐reported outcomes and sensor data), coaching, and navigation, is depicted.

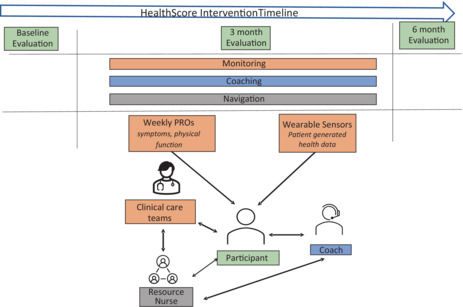

## 1 INTRODUCTION

Cancer and its treatment greatly impact the physical and psychological functioning of patients.^1^ Individuals with cancer experience declines in physical function and subsequently may have decreased treatment tolerance, higher health service utilization, and shorter survival.^2^, ^3^, ^4^ Despite interdisciplinary agreement on physical activity guidelines for cancer patients,^5^ approximately only 30‐47% of patients meet these guidelines.^6^, ^7^, ^8^

Existing interventions focused on improving physical functioning in cancer survivors have limitations including generalizability, alignment with patient goals, and cost. Most interventions have been developed for use in breast, prostate, and colorectal cancer survivors,^9^, ^10^ and may not be generalizable to individuals undergoing active cancer treatment or to survivors of other types of cancer such as hematologic cancers. Interventions have generally focused exclusively on nutrition and/or exercise,^11^ which may not always align with patient goals and do not routinely address barriers to physical activity such as symptom burden.^12^

In light of the limitations of existing interventions, we developed a novel theory‐based intervention utilizing patient‐generated health data (PGHD) called the HealthScore Health Coaching Program (HealthScore). HealthScore focuses on overall health optimization for persons impacted by cancer, through the delivery of weekly curricular‐based participant‐driven coaching sessions, collection of person‐reported outcomes (PROs), and physiologic‐based patient‐generated health data to navigate participants towards comprehensive supportive services to facilitate their vision of best health. With patient input, we developed a comprehensive intervention program that included a primary focus on physical function and quality of life, wellness, and self‐efficacy. PROs and digital monitoring of PGHD in clinical oncology practice have been shown to reduce health care utilization and improve quality of life and survival.^13^, ^14^, ^15^ However, there is less research integrating digital monitoring of PROs and reporting results for use in clinic‐based workflows. In addition, PRO digital monitoring is not routinely conducted by oncology clinical care teams, and especially not when oriented towards proactive health optimization.

Our purpose was to (1) assess the feasibility and acceptability of the intervention and (2) describe changes from baseline to 3 and 6 months of the targeted primary (physical function) and secondary patient‐reported outcome changes of a single‐arm pilot of HealthScore at the North Carolina Basnight Cancer Hospital.

## 2 METHODS

HealthScore is based on the principles of self‐determination theory (autonomy, relatedness, and competency)^16^ and the transtheoretical model of behavior change^17^ and utilizes motivational interviewing techniques.^18^ The intervention includes consistent attention to physical function, quality‐of‐life, wellness, self‐efficacy, and symptom management. Participants decide which domains of health they wish to prioritize and optimize, and they work with their coaches through weekly and milestone coaching sessions. Physical and PRO assessments help to inform participant‐driven goals and monitor achievement. Within these areas, participants can further prioritize specific health aspects such as exercise, nutrition, sleep, stress management, meaning‐making, relationships and communication, spirituality, and others. The intent of this health optimization approach is to promote participant autonomy and self‐efficacy for decisions and behaviors that will build resilience, improve well‐being, and mitigate the impact of underlying disease and treatment.

### 2.1 Intervention description

**FIGURE 1:**
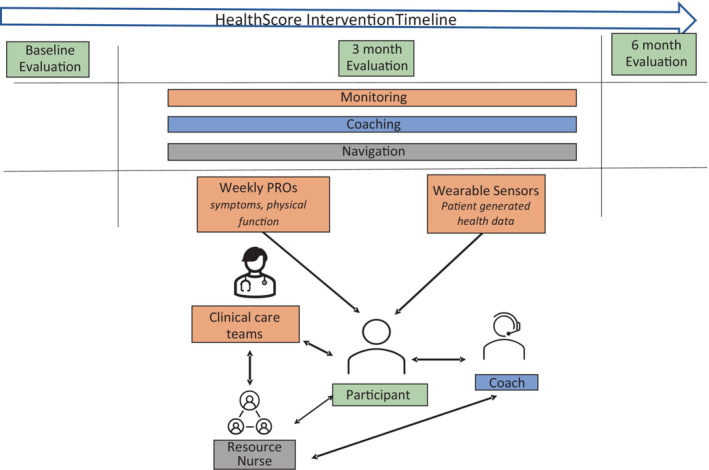
Schematic of the health coaching intervention components

HealthScore was established as a pilot, single‐arm study. Recruitment began in May 2020, during the early months of the COVID‐19 pandemic, and so, all study procedures including consent, FitBit orientation, onboarding to the survey platform, physical and cognitive performance testing, coaching, and navigation were performed remotely. The 4‐m walk test for gait speed was included as it was deemed feasible to have participants conduct remotely. PRO assessments were completed via an institutional software platform with additional survey administration capabilities, a system called PRO‐Core. A secure program software portal was developed for administrators and coaches, and all PGHD was linked to the portal (by API from Fitbit accounts, and by direct connection to PRO‐Core) for continuous monitoring, data visualization, report generation, coaching documentation, and goal setting. Figure 1 provides a schematic of the intervention design.

### 2.2 Physical activity trackers

Participants received a Fitbit with features that included heart rate tracking, step count, and active minute monitoring. Participants were trained in how to use and sync the tracker. Study staff were available for troubleshooting as needed.

### 2.3 Measures

Sociodemographic data were collected at baseline, in addition to remotely administered cognitive testing (Montreal Cognitive Assessment (MoCA)^19^) and physical performance testing (6‐min walk test and gait speed). Comprehensive electronically administered survey packets were administered at baseline, 3 months, and 6 months, in addition to a smaller set on a monthly basis, and a still smaller set on a weekly basis. These packets contained validated instruments as described in Table S1, which covered domains including physical health, anxiety, depression, social support, unmet needs, and a variety of other topics. Weekly measures are further described in the coaching description below. Participant measures of feasibility and acceptability^20^ of both the coaching calls and Fitbit tracking were assessed at the end of the intervention. Interviews were conducted at the study exit to elicit participant perspectives on the feasibility and acceptability of the intervention, and to guide further program development.

### 2.4 Coaching

Coaching curriculum in the pilot included a comprehensive suite of health domains oriented around physical function, quality‐of‐life, wellness, self‐efficacy, and symptom management. Specific topics that were addressed included physical activity, nutrition, sleep, stress management, meaning‐making, relationships and communication, and spirituality. Coaches were trained in motivational interviewing techniques to ensure that coaching calls were participant‐driven and goal‐oriented. Coaching training manuals and videos were developed, and additional coaches were recruited including volunteer health professionals and lay individuals. In total, the coaching staff was comprised of two permanent cancer center staff members who also coached participants, in addition to 12 volunteer coaches. Coaches were trained systematically in a series of didactic presentations in the transtheoretical model of behavior change, motivational interviewing techniques, coaching foundations, cancer center resources, exercise guidelines, and prescription, vetted resources corresponding to other health domains, and pathways for escalating participant‐identified concerns that were beyond coaching expertise to the participant's primary clinical team, the HealthScore's medical directors, or the HealthScore resource nurse. Coaches were required to complete a set number of practice sessions with a mock patient and listen to a set number of real coaching calls. The trainer, a national board‐certified health and wellness coach and exercise physiologist, listened to the lay coaches' calls to ensure the appropriate use of skills, provide feedback, and provide real‐time support as needed. At baseline, coaches engaged with each newly enrolled participant in a “microsession,” in which participants were asked to describe their vision of their best health and were guided to prioritize domains of health that were meaningful to them.

During coaching calls, participants were guided in setting goals that were specific, measurable, attainable, relevant, and time‐bound (SMART). Each week, coaches used information from the participant's Fitbit, PRO responses, and coaching call conversations to guide the development of SMART goals that were subsequently sent to the participant by email after the weekly coaching call. Triggers and alerts were developed for all PROs so that responses that exceeded certain thresholds were translated by the HealthScore resource nurse to notifications or referrals to primary oncologists or other cancer center services. In conjunction with personnel in different cancer center supportive care services, the team developed pathways to connect appropriate participants to palliative and supportive care, nutrition, chronic pain management, neuropsychological testing, psychological counseling or medication management, physical therapy, pharmacy, chaplaincy, and patient and family resource center. Coaches and the resource nurse were notified by the program software portal when alerts were generated so that they could follow‐up with participants within one week of each alert to ensure that participant needs had been addressed. The software portal included data visualizations for each participant that represented weekly physical function (modified PROMIS Physical Function 8B,^21^ referred to as the “HealthScore”) weekly individual symptom responses (symptoms from the patient‐reported outcomes version of the common terminology criteria for adverse events [PRO‐CTCAE] symptoms,^22^ with multi‐attribute symptoms collapsed into a single symptom score), and weekly symptom burden (each individual symptom score from each week summed to represent a weekly aggregate symptom burden). During the pilot, the software portal was also configured to generate weekly reports containing weekly participant data and trends, as well as coaching notes; these reports could be cut and pasted into the electronic health record, forwarded to the HealthScore medical director for attestation, and made available to other clinicians or cancer personnel from whom participants might receive care.

### 2.5 Interdisciplinary team meetings

Weekly team meetings among the investigators, project manager, study coordinator, and resource nurse were held during the pilot and attended by coaches and additional representatives from oncology, palliative care, nursing, program management, and project leaders. The purpose of the meeting was to update the team on specific participants at key intervention milestones, as well as discuss coaching and symptom alert pathways. Participants were stratified according to three risk levels (high, intermediate, low) based on the following criteria, unplanned hospitalization, and emergency department visits (low none; intermediate 1; high >2), physical function based on the PROMIS PF scale (low >45; intermediate <45; high <45 + physical therapy), number of symptom alerts and referrals made (low 0/week; intermediate 1‐3/week; high >3/week), medical risk based on treatment and disease status (low‐ not on treatment, on surveillance; intermediate‐ advanced cancer/active treatment, but with stable disease; high‐ advanced cancer/active treatment and deterioration in clinical status) and availability of psychosocial support (low lives with caregiver or has good support network; high‐ lives alone or high caregiver burden or financial distress or remote rural location) (See Table S2). During each meeting, a subset of participants was presented to the group at which time each participant's story was summarized, longitudinal data were reviewed, the risk for future adverse events was predicted, and any ongoing issues were discussed. The team provided input and suggestions to help meet ongoing participant needs, and lessons from each participant's experience were brought back to iteratively improve the intervention for future participants.

### 2.6 Outcomes

Outcomes were collected at baseline, 3 months, and 6 months postenrollment. Feasibility was measured by the number of coaching calls completed out of the total possible for the weeks a participant was enrolled. Acceptability of coaching calls and feasibility of wearing the Fitbit were measured from the perspective of participants using two 4‐item validated implementation outcome measures (range: 0‐5) and were measured at the six‐month survey time point.^20^ To investigate whether intervention participants experienced improvements in health, survey measures were collected at the three milestones. The primary preliminary outcome measure was participant‐reported physical function, using PROMIS PF 8B, given its validation in previous diverse cancer samples.^21^ In addition, several secondary outcome measures were collected to capture the quality‐of‐life domains critical for functioning, using PROMIS Cognitive Function, Emotional Support, Depression, Anxiety, and Social Isolation.^23^, ^24^ In addition, individual PRO‐CTCAE symptom items were summed each week to calculate a cumulative symptom burden measure, based on data suggesting a relationship between elevated symptom burden and reduced physical function.^25^ Finally, the Lorig 6‐item self‐efficacy measure (range: 1‐10) was also collected at all three milestones.^26^ Daily steps were captured and averaged at baseline (daily steps averaged baseline week and week 1), 3 months (averaged at weeks 14, 15, and 16), and 6 months (averaged at weeks 24, 25, 26). Finally, a qualitative, semi‐structured exit interview was conducted after participants completed the intervention. Interviews focused on the perception of intervention components, perceived short‐ and long‐term benefits of the intervention, and recommendations for changing the intervention. Interviews were audio recorded, transcribed, and representative experience quotes were identified. The Institutional Review Board of the University of North Carolina at Chapel Hill approved the study (IRB# 20–0051). All patients provided written informed consent.

### 2.7 Statistical analysis

Summary statistics, including proportions for categorical variables, mean and standard deviation (SD) for numerical variables, were calculated on all acceptability, feasibility, and primary outcome measures. For key outcomes like acceptability and feasibility, associated 2‐sided Clopper–Pearson exact 95% confidence intervals (95% CI) were also calculated. The study is registered at ClinicalTrials.gov (identifier NCT04923997).

## 3 RESULTS

### 3.1 Feasibility

Between May 2020 and March 2022, 185 potential participants were referred by clinicians, screened via the EHR for possible eligibility, and 163 approached via email or phone. Of those, 22 were ineligible, and of the remaining 141, 106 responded, 70 enrolled and were consented (66% of those who responded), and 50 completed the intervention and comprised the final pilot sample (71% of those who initiated). Of those that did not complete the intervention, four withdrew due to not having enough time, treatment burden, or disease progression; 12 were lost‐to‐follow‐up; and four died before interview completion.

Approximately 32% of the pilot sample were between ages 15 and 39 at study enrollment, 42% ages 40‐64, and the remaining 26% ages 65 and over (Table 1). There were slightly more women than men participants (52%). Approximately 84% of the participants identified as non‐Hispanic White, followed by 10% Black or African American. Most participants (78%) had a least an Associate's degree or higher. About 34% of the sample were employed full‐time. The cancer site distribution was mostly hematologic cancer (46%) and breast cancer (28%), and the remaining cancer sites (19%) were sarcoma, genitourinary, lung, head, and neck, gynecologic, and melanoma.

**TABLE 1:** Healthscore phase 2 participant demographics, n = 50

Variable	Frequency	Percent
Demographics		
Age		
15‐39	16	32
40‐64	21	42
65+	13	26
Average (mean [SD])	51 (15.8)	
Sex		
Male	21	42
Female	29	58
Race		
White	44	88
Black or African American	5	10
Asian American	1	2
Ethnicity		
Hispanic/Latinx	1	4
Non‐Hispanic/Non‐Latinx	49	98
Education		
9th‐12th grade (no diploma)	1	2
High school graduate or equivalent (GED)	2	4
Some college (no degree)	6	12
Vocational or Associate's degree	7	14
Bachelor's degree	18	36
Higher than Bachelor's degree	14	28
*Missing*	2	4
Employment		
Employed full‐time	18	36
Employed part‐time	6	12
Unemployed	3	6
On disability	7	14
Retired	10	20
Homemaker/Student/Volunteer	6	12
Marital/partner status		
Single, never married	8	16
Married or living with a partner	36	72
Divorced	3	6
Widowed	1	2
*Missing*	2	4
Household Income		
Less than $20 000	4	8
$21 000‐$39 999	4	8
$41 000‐$59 999	8	16
$61 000‐$79 999	7	14
$80 000‐$99 999	4	8
$100 000 or more	20	40
*Missing*	3	6
Cancer site		
Hematologic	23	46
Breast	14	28
Sarcoma	2	4
Genitourinary	2	4
Lung	2	4
Head and neck	1	2
Gynecologic	2	4
Melanoma	4	8

### 3.2 Acceptability

We present findings on participants who completed the full 6‐month single‐arm pilot. Intervention engagement was high; 86% (95% CI (0.73, 0.94)) participated in at least 75% of weekly coaching calls, and 75% with 95% CI (0.61, 0.86) completed at least half of the weekly physical function and symptom assessments. Milestone assessment completion was high, with 96%, 78%, and 100% of baseline, 3‐month, and 6‐month surveys completed, respectively. Participant satisfaction overall was high, with an average of 4.8 (SD = 0.90, range 0‐5) on the acceptability of coaching calls and 4.7 (SD = 0.51, range 0‐5) on ease of using the Fitbit.

### 3.3 Preliminary outcome data

**FIGURE 2:**
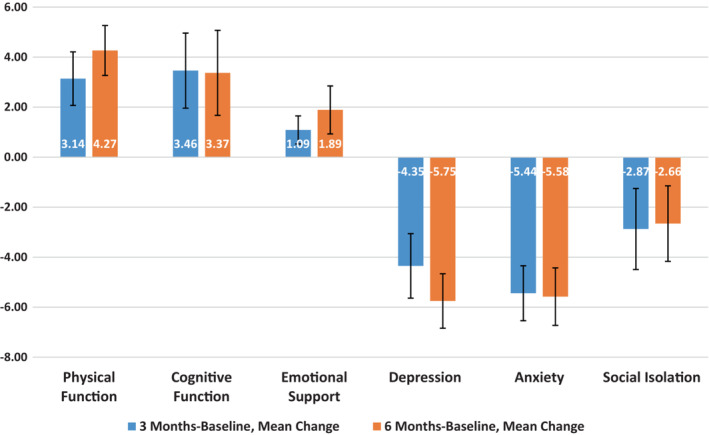
Mean changes in patient‐reported outcomes, using PROMIS measures, from baseline to 3 months (blue) and 6 months. Error bars shown

Table 2 and Figure 2 show preliminary results from targeted outcomes. PROMIS Physical Function scores rose an average of 3.1 points (SE = 1.1) from baseline to 3 months, and 4.3 (SE = 1.0) from baseline to 6 months, or almost one‐half a standard deviation from baseline to 6 months. Secondary PROMIS outcomes also showed positive results, with gains in cognitive function and reductions in depression, anxiety, and social isolation. In addition, symptom burden also decreased on average by 4.3 points (SE = 0.50, Range 0‐29) from baseline to 3 months, and 4.1 points from baseline to 6 months (SE = 0.52). Self‐efficacy improved by 0.65 points (SE = 0.24, Range 0‐10) from baseline to 3 months and 1.1 points (SE = 0.28) from baseline to 6 months. Average daily step counts increased from baseline (3640) to 3 months (6845) and 6 months (7053). Four‐meter walk times also shortened, by almost a second, from baseline (4.65) to 3 months (3.78). At 6 months, walk times increased slightly from the 3 months timepoint to 4.06 s.

**TABLE 2:** Preliminary outcome estimates from participants in HealthScore Phase 2 single‐arm pilot

	Baseline			3 months			6 months			Mean change baseline to 3 months			Mean change baseline to 6 months		
Outcome (Range)	N	Mean	SD	N	Mean	SD	N	Mean	SD	N	3 Months‐Baseline, Mean Change	SE	N	6 Months‐Baseline, Mean Change	SE
Physical function^a^	50	42.47	7.25	47	46.09	9.65	45	47.10	9.70	47	3.14	1.07	45	4.27	1.00
Cognitive function^a^	47	44.18	11.73	39	48.11	10.80	50	47.30	10.57	37	3.46	1.50	47	3.37	1.70
Emotional support^a^	47	53.81	9.56	38	55.45	9.02	49	55.78	8.87	36	1.09	0.56	46	1.89	0.96
Depression^a^	47	53.80	7.71	39	48.98	9.43	49	48.37	9.70	37	−4.35	1.29	46	−5.75	1.09
Anxiety^a^	47	54.09	9.04	39	48.64	8.77	50	49.04	9.36	37	−5.44	1.10	47	−5.58	1.15
Social isolation^a^	45	47.92	9.07	39	44.08	9.25	50	44.44	10.57	35	−2.87	1.62	45	−2.66	1.51
Symptom burden (0‐29)	40	11.20	6.05	43	6.90	5.51	45	6.57	5.62	36	−4.29	0.50	36	−4.12	0.52
Self‐efficacy (0‐10)	46	6.80	1.89	38	7.74	1.73	49	7.83	2.09	36	0.65	0.24	46	1.09	0.28
FitBit recorded average daily number of steps	36	6032	3640	45	6845	4506	43	7053	4405						
4 m walk average time (seconds)	24	4.65	1.48	22	3.78	1.07	20	4.06	1.33						

^a^ PROMIS measures, scale 0‐100.

### 3.4 Participant Experiences

We completed 46 (94%) patient interviews at the 6‐month timepoint. Participants who completed the interview reported high levels of satisfaction with the intervention (93%, 95% CI (0.82, 0.98)), a desire to continue if it were an option (81%, 95% CI (0.67, 0.91)), and that they had a meaningful experience with their coach (96%, 95% CI (0.86, 0.99)). Participants who did not want to continue with their coach reported feeling they had the tools to move forward on their own and were appreciative of the support HealthScore provided. Participant responses when probed about their experience were overwhelmingly positive. The one suggestion made was to try to cut down the overall number of survey items at the milestone interviews. The following are representative statements from program completer exit interviews: “Now everything is easier. I can do things with my family I couldn't before.” “The program helped me feel better mentally and physically. I got my ‘old energy’ back. Exercise helped me transition off my anxiety medications.” “I notice a difference in my strength and energy which helps me as a teacher and as a mom.” “I've gotten back to taking care of my kids and to a place that is a good new normal—it's only going to get better.”

## 4 DISCUSSION

We have developed and piloted the HealthScore health coaching intervention to optimize health for individuals living with cancer in a way that is patient‐centered and focused on the autonomy, values, and preferences of individual participants. HealthScore has been built to improve upon existing constraints of supportive care interventions, including limitations in generalizability, lack of alignment with patient goals, and lack of potential scalability due to cost. We have built HealthScore using electronic patient‐reported outcome instruments, remotely administered physical and cognitive testing, continuously obtained PGHD from wearable health Fitbits, weekly coaching and health goal setting, and clinically integrated navigation. Using this virtual and technology‐enabled approach, we have built an intervention that can meet patients where they are, respecting and maximizing time spent at home and away from the health care setting.^27^ Our main findings from the pilot study were that the six‐month coaching program was feasible and acceptable to participants, based on satisfaction scores and exit interview findings. Though we do not yet have a control group comparison, we found preliminary evidence for the preservation of physical function, stabilization of symptom burden, and improvements in depression and anxiety symptoms for participants who completed the 6‐month intervention.

The HealthScore pilot had favorable feasibility findings, comparable to other recent studies, with initiation rates at 66% and 6‐month completion rates at 71%. A feasibility trial of a three‐month digital health coaching program for men treated for prostate cancer showed feasibility at 72% (threshold 60%).^28^ Another feasibility trial of an 8‐week health coaching mobile app for esophageal cancer patients showed 70% use rates until the end of the trial.^29^ Finally, a third study protocol designed to improve fitness and patient‐reported outcomes among breast cancer patients using technology‐enabled health coaching aims to recruit at least 40% of eligible, contacted patients and retain at least 75% through a 6‐month assessment (with 70% of intervention components completed).^30^

### 4.1 Research and clinical implications

We believe that there are several features of HealthScore that build upon the existing literature and hold promise for further development and testing. First, the intervention is participant‐centered; HealthScore participants chose domains of health they wished to prioritize and are empowered by coaches to identify realistic ways to meet these goals and provided PRO and patient‐generated health data to guide the navigation of supportive cancer services to meet their needs. This participant‐centeredness may have contributed to the high acceptability of the intervention, consistent with prior research about patient preferences regarding navigation and digital medicine programs.^31^, ^32^ Second, HealthScore favors proactive health optimization based on both PRO and PGHD data capture, which resonates with prior literature related to resilience‐based^33^, ^34^ and prehabilitative^35^ interventions, and also to geriatric assessment‐guided approaches to address underlying vulnerability.^35^ In these approaches, upstream determinants of health outcomes are identified and managed so that participants develop physical and psychological reserves that allow them to withstand later disease‐ or treatment‐related toxicities. We hypothesize that in using this approach, we may be better able to preserve or increase function and quality of life, while improving participant coping and decision‐making skills, than if we had adopted methods focused exclusively on recognizing and responding to impending emergencies. Finally, we believe that the intervention illustrates the enthusiasm and potential of lay volunteer coaches to deliver impactful health interventions for individuals living with cancer. The effective use of lay health workers in cancer health care delivery interventions has been demonstrated previously,^36^ and we believe using a combination of a certified health coach trainer and a cohort of lay coaches balances patient safety and fidelity with potential scalability if efficacy is demonstrated in the planned trial.

### 4.2 Study limitations

We recognize several limitations that will need to be addressed for our intervention to become more successful. Adherence to assessments remains a challenge as it does for all PRO/PGHD utilizing programs in cancer care. However, we have reviewed experience with PRO implementation in cancer clinical trials,^37^ and have found reminders from coaches, elimination of redundant or less useful questions or instruments, and providing data back to participants to be successful strategies that have improved our adherence over time. We have also noted opportunities to increase the efficiency of alert‐ and referral‐based pathways to navigate cancer center resources to participants and have found that the incorporation of a formal resource nurse role has greatly improved our ability to do this successfully. Scalability of interventions such as HealthScore that utilize existing staff will likely require the identification of central staffing strategies for retention. In addition, our future efficacy trials will require consideration of cultural adaptations,^38^ reach of individuals with lower digital health literacy, reaching those with lower digital health literacy,^39^ and, and those without reliable internet access.^40^ For future versions of the intervention program, we have created a tool to monitor sociodemographics, including race and ethnicity, rurality, and the social vulnerability index score of the zip code potential participants reside in, with the intent to increase outreach to a more diverse sample of participants.

### 4.3 Future directions

Based on findings from our single‐arm pilot of the HealthScore intervention, we believe that we are now ready to test the efficacy of HealthScore for improving physical function when compared to an appropriate control group. We have recently implemented an initial pilot randomized, two‐arm study of the HealthScore intervention in individuals living with advanced or metastatic cancer available in English and Spanish. In a subsequent fully powered efficacy trial, we plan to study maintenance or improvement of physical function as our primary outcome but will also examine differences in physical activity, psychological symptoms, health‐related quality‐of‐life, self‐efficacy, and acute care events. We will seek to identify participant‐level factors associated with differential intervention effects and those without reliable internet access. Data from the initial efficacy study will allow us to further develop predictors of physical function (e.g., “digital biomarkers”),^41^ using concurrent symptoms and Fitbit data, which in turn will lead to further coaching intervention improvements.

During the rise of virtual health care delivery services during the COVID‐19 pandemic, we have developed, implemented, and enhanced a health coaching intervention for individuals living with cancer. We plan to formally test our intervention and to improve it further so that it can best meet the needs of our participants while serving as an example for other technology‐enabled coaching programs for this population.

## AUTHOR CONTRIBUTIONS

**William A Wood:** Conceptualization (equal); funding acquisition (lead); investigation (lead); methodology (equal); supervision (lead); writing – original draft (equal); writing – review and editing (equal). **Carly Bailey:** Conceptualization (supporting); data curation (supporting); investigation (supporting); project administration (equal); supervision (equal); writing – original draft (supporting); writing – review and editing (supporting). **Brianna Castrogivanni:** Data curation (supporting); investigation (supporting); project administration (supporting); writing – review and editing (supporting). **Diana Mehedint:** Conceptualization (supporting); investigation (supporting); methodology (supporting); writing – review and editing (supporting). **Ashley Bryant:** Conceptualization (supporting); investigation (supporting); methodology (supporting); writing – review and editing (supporting). **Kyle Lavin:** Conceptualization (supporting); investigation (supporting); methodology (supporting); writing – review and editing (supporting). **Xianming Tan:** Conceptualization (supporting); formal analysis (equal); investigation (supporting); writing – original draft (supporting); writing – review and editing (supporting). **Jaime Richardson:** Investigation (supporting); project administration (supporting); resources (supporting); writing – review and editing (supporting). **Yiqing Qian:** Data curation (supporting); formal analysis (supporting); writing – original draft (supporting); writing – review and editing (supporting). **Kelly R. Tan:** Conceptualization (supporting); investigation (supporting); methodology (supporting); project administration (supporting); supervision (supporting); writing – original draft (supporting); writing – review and editing (supporting). **Erin Kent:** Conceptualization (equal); data curation (lead); formal analysis (lead); investigation (equal); methodology (lead); project administration (equal); supervision (equal); writing – original draft (lead); writing – review and editing (lead).

## FUNDING INFORMATION

KRT received funding from the National Cancer Institute (T32CA116339).

## CONFLICT OF INTEREST

WAW reports research funding from Pfizer and Genentech, consulting for Teladoc, and is an advisor with equity for Koneksa Health.

## Supporting information


Table S1.

Table S2.
