# Supplementary material for: Piloting HealthScore: Feasibility and acceptability of a clinically integrated health coaching program for people living with cancer
Source: Cancer Med. 2023 Jan 16;12(7):8804–14. doi: 10.1002/cam4.5625 (PMC10134320; doi:10.1002/cam4.5625)
Supplement: Supplementary file 1 — Table S1. [file CAM4-12-8804-s001.docx]

| **Supplemental Table 1.** Assessments and schedule for both study phases. | | |
| --- | --- | --- |
| **Assessment** | **Developmental Phase** | **Single-Arm Pilot** |
| Symptom Monitoring | W | W |
| Physical Function (PROMIS) | W | W |
| Falls Question | - | Mo |
| Self-reported Performance Status | - | Mo |
| PROMIS Global Health- 10 item | - | Mo |
| PROMIS Cognitive Function | MS | Mo |
| PROMIS Depression | MS | Mo |
| PROMIS Anxiety | MS | Mo |
| PC-PTSD/PCL5 | - | Mo |
| PONS (nutrition) | - | Mo |
| PGSGA (nutrition) | MS | - |
| AHC HSRN Screening Tool* | - | Mo |
| Self-Reported Medication Adherence | - | Mo |
| Self-reported Performance Status | MS | MS |
| Self-reported exhaustion/activity )** | - | MS |
| NHANES Physical Activity Survey | - | MS |
| Instrumental Activities of Daily Living (Lawton) | MS | MS |
| Montreal Cognitive Assessment (MOCA) | - | MS |
| Lorig Self Efficacy | MS | MS |
| AAQ-II* | - | MS |
| Herth Hope Index* | - | MS |
| EQ-5D-5L | MS | MS |
| PROMIS Emotional Support | MS | MS |
| PROMIS Social Isolation | MS | MS |
| Health Behaviors | MS | MS |
| Advance Directives | MS | MS |
| Financial toxicity screening  (including COST-FACIT) | - | MS |
| Ego Resilency | MS | - |
| PROMIS- Ability to participate in social roles and activities | MS | - |
| Abbreviations: E-Pro Assessments (W=weekly, Mo=monthly, MS=milestone (baseline, 3 months (phase 2 only), EOS)  *Replaced in participants aged 15-39 years with surveys listed below.  -  Herth Hope Index and AHQ replaced with Child and Youth Resilience Survey  - AHC HSRN screening tool replaced with AYA unmet needs survey | | |
