# Supplementary material for: Piloting HealthScore: Feasibility and acceptability of a clinically integrated health coaching program for people living with cancer
Source: Cancer Med. 2023 Jan 16;12(7):8804–14. doi: 10.1002/cam4.5625 (PMC10134320; doi:10.1002/cam4.5625)
Supplement: Supplementary file 2 — Table S2. [file CAM4-12-8804-s002.docx]

**Supplemental Table 2.** Blank risk stratification template for prioritizing participants in weekly interdisciplinary team meetings.
